# Giant vulvar verruciform xanthoma in a child: a rare case report and literature review

**DOI:** 10.3389/fmed.2026.1774843

**Published:** 2026-04-22

**Authors:** Yan Xia, Ying Liu, Hongyan Jiang, Hongsheng Xia, Bin Dong

**Affiliations:** 1 Department of Ultrasonography, Hangzhou Third People's Hospital, Hangzhou Third Hospital Affiliated to Zhejiang Chinese Medical University, Hangzhou, China; 2 Cancer Center, Department of Ultrasound Medicine, Zhejiang Provincial People’s Hospital (Affiliated People’s Hospital), Hangzhou Medical College, Hangzhou, Zhejiang, China; 3 Department of Dermatology, Hangzhou Third People’s Hospital, Hangzhou Third Hospital Affiliated to Zhejiang Chinese Medical University, Hangzhou, China

**Keywords:** child, female, ultrasonographic findings, verruciform xanthoma, vulva

## Abstract

Verruciform xanthoma (VX) is a rare benign mucocutaneous proliferative lesion that primarily affects the oral mucosa and, in rare cases, the genital mucosa and skin. Due to its uncommon occurrence and varied, non-specific clinical manifestations, the condition is prone to misdiagnosis and missed diagnosis. This report introduces an even rarer case of pediatric vulvar VX, providing a detailed interpretation and analysis of the ultrasonographic features of this condition. Furthermore, in conjunction with previous literature, this report summarizes and analyzes the currently reported vulvar VX cases, aiming to obtain effective diagnostic and therapeutic information, enhance preoperative diagnostic accuracy, and improve patient prognosis.

## Introduction

Verruciform xanthoma (VX) is a rare benign proliferative lesion that occurs on the skin and mucous membranes, with only 20% involving the vulva or skin (1), and is more commonly seen in areas such as the penis, scrotum, anus, and vulva. The incidence of VX in the female perineal region is low and is particularly rare in children. Lesions are usually solitary, with multiple or multi-site involvement being rare (2).

Due to the rarity of this disease, the diagnostic challenge it poses, its extracutaneous manifestations, and potential delays in diagnosis, the condition is often associated with poor prognosis. Herein, this study reports a pediatric case of giant vulva VX accompanied by verrucous tumor of the limbs and trunk. The study analyzed the ultrasonographic features and summarized the clinical significance of ultrasound in the diagnosis and treatment of VX. The study also discussed the differential diagnosis from other skin diseases, including the unique clinical and histological features of each condition. Additionally, the study retrospectively summarized and analyzed the characteristics of previously reported cases. The patient was diagnosed with vulvar VX and achieved a positive outcome.

## Case description

### Clinical presentation

**Figure 1:**
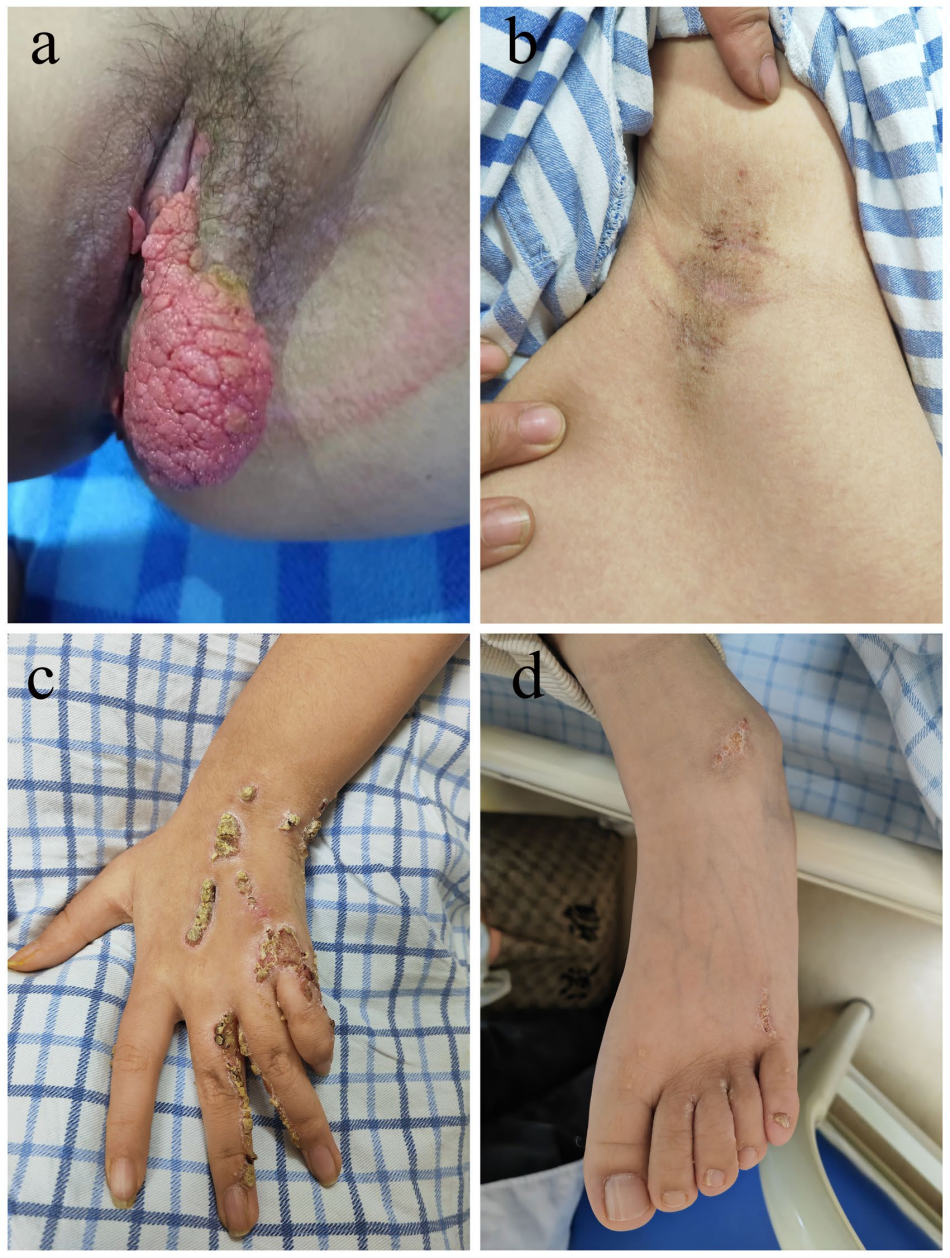
Physical examination, appearance of giant vulva VX in a pediatric patient. **(a)** The skin mass near the labia majora of the perineum measures approximately 10 cm × 5 cm. **(b)** A similar but relatively small lesion on her left axilla. **(c)** A similar but relatively small lesion on her left hand. **(d)** Similar but relatively smaller lesions on her feet.

The patient, a 12-year-old girl, was admitted to the hospital with a perineal skin mass of unknown cause that had been present for 8 years. Initially, the lesion was small and of medium consistency, without specific skin manifestations such as blisters, bullae, erosions, ulcers, bleeding, exudation, scaling, or peeling. She also had no symptoms of discomfort such as chills, fever, local redness, swelling, or pain. During this period, the condition was not taken seriously. Subsequently, the mass gradually enlarged, and the patient presented to our hospital for further evaluation and treatment. Specialized physical examination revealed a pink, cauliflower-like skin mass near the labia majora of the perineum, measuring approximately 10 cm × 5 cm (Figure 1a). The lesion had poorly defined borders and an irregular and elevated surface, and no obvious blisters, pustules, erosions, or ulcers were observed. Additionally, similar but relatively smaller lesions were present on the patient’s left hand, axillae, perineum, and both feet (Figures 1b–d). Laboratory findings, including complete blood count, liver function tests, renal function tests, coagulation profile, lipid profile, HIV antibody screening, and the Venereal Disease Research Laboratory (VDRL) test, were all unremarkable.

### Ultrasonographic findings

**Figure 2:**
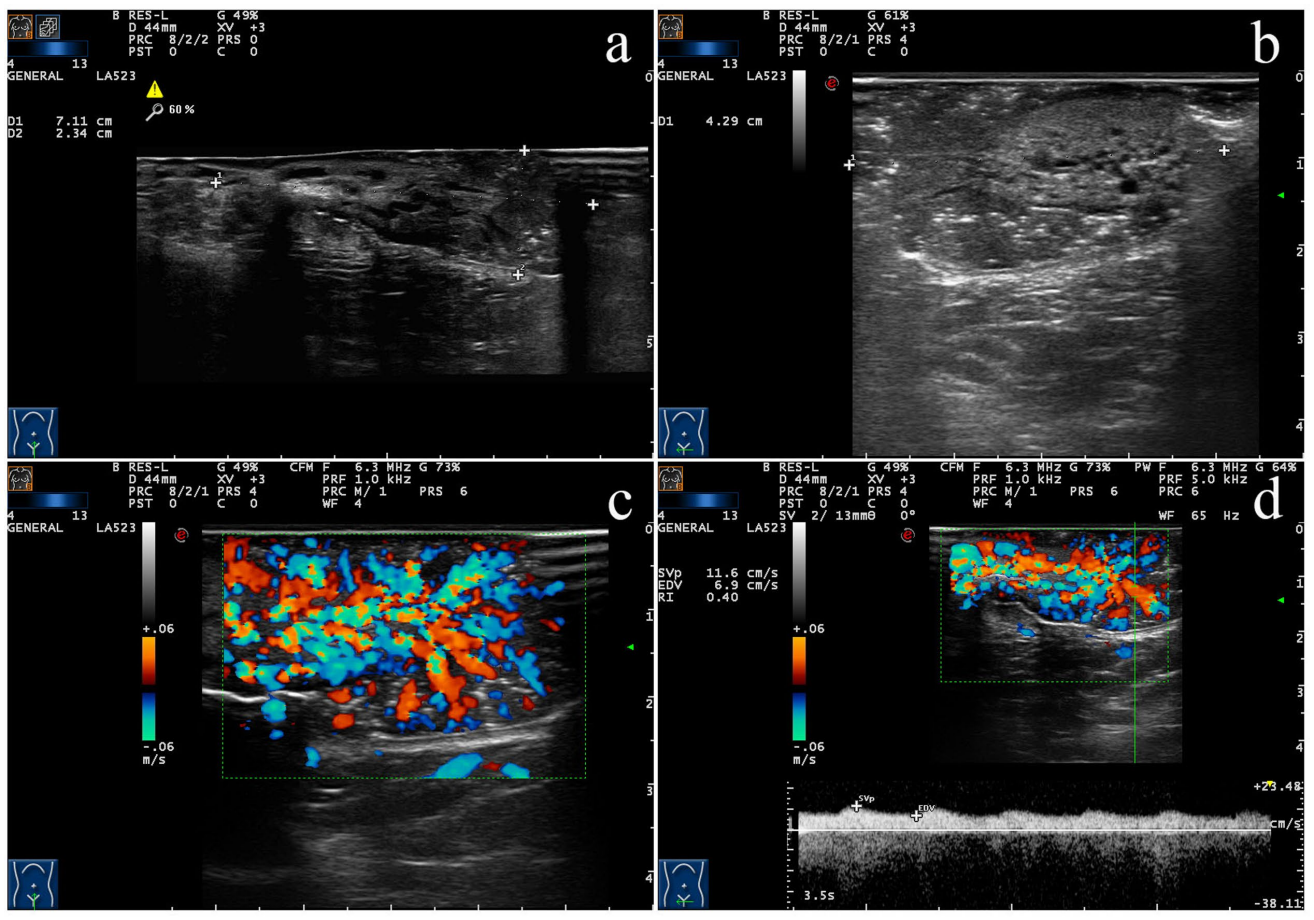
Ultrasound findings of giant vulva VX in a child. **(a)** The maximum cross-sectional image of the lesion under two-dimensional (2D) ultrasound. **(b)** Image of the perpendicular cross-section to the maximum cross-section of the lesion under 2D ultrasound. Multiple punctate hyperechoic foci and several strip-like anechoic areas were observed within the hypoechoic lesion. **(c)** CDFI demonstrated abundant blood flow signals inside the mass, presenting a “fiery sea sign-like” pattern. **(d)** Pulsed Doppler ultrasound reveals low-velocity and low-resistance blood flow within the lesion.

Ultrasound examination revealed a hypoechoic mass measuring approximately 7.1 cm × 2.3 cm × 4.3 cm beneath the vulvar mucosa (Figures 2a,b). The mass exhibited ill-defined borders and an irregular shape, with heterogeneous internal echogenicity. Multiple punctate hyperechoic foci and several strip-like anechoic areas were observed within the lesion. The color Doppler flow imaging (CDFI) demonstrated abundant blood flow signals inside the mass, presenting a “fiery sea sign-like” pattern (Figure 2c). The pulsed Doppler ultrasound measured a resistance index of 0.40 (Figure 2d). The ultrasound findings suggest a vulvar mass with a rich blood supply.

### Pathology

**Figure 3:**
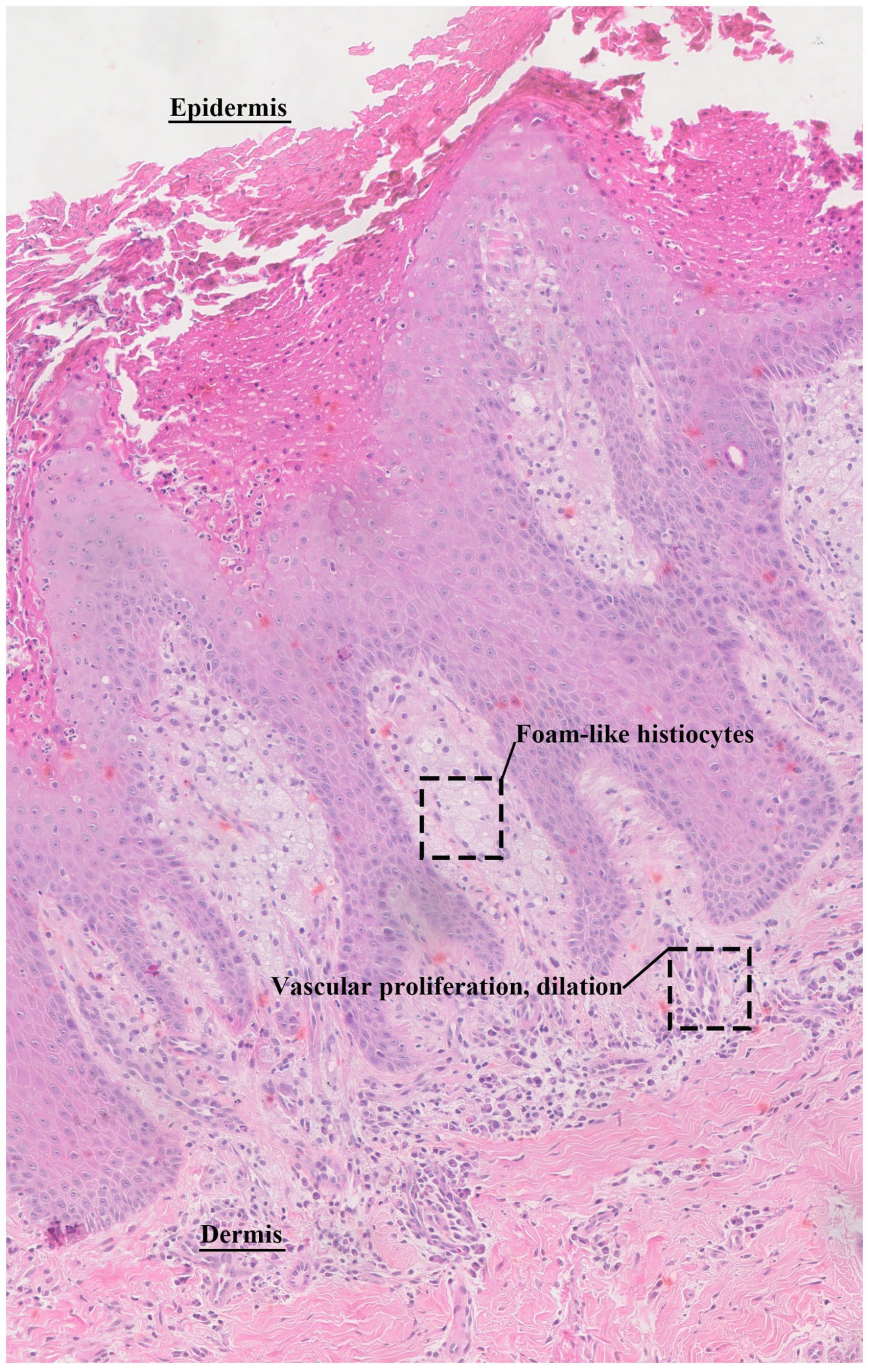
Hematoxylin and eosin (HE) stained the pathological image of the lesion. The dermal papillae showed extensive infiltration by foamy histiocytes, accompanied by perivascular sparse lymphocytes and numerous plasma cells. The superficial dermis showed vascular proliferation, dilation, and congestion.

After completing all necessary examinations, the patient underwent staged excision of the perineal skin lesion under general anesthesia, combined with harvesting and transplantation of a pedicled axial pattern flap. Postoperative pathology (Figure 3) revealed epidermal acanthosis with elongated rete ridges. Neutrophils were scattered or aggregated within the stratum corneum and crypts. The dermal papillae showed extensive infiltration by foamy histiocytes, accompanied by sparse perivascular lymphocytes and numerous plasma cells. The superficial dermis showed vascular proliferation, dilation, and congestion. These findings are consistent with the pathological changes of VX.

### Follow-up

The patient has been followed up for approximately 3 years after surgery. Based on the recent follow-up results, no lesion recurrence was observed in the surgical area of the vulva. The lesions at other sites were not surgically treated at that time. During subsequent regular follow-ups, the growth rate of these lesions has remained relatively slow, with no significant change in size. Currently, the patient has no plans for further treatment.

## Literature review

The study conducted a search of the PubMed, China National Knowledge Infrastructure, Wanfang Data, and Embase databases for articles published from 1979 to 2026. A total of 38 vulvar VX cases in 26 reports (3–28) were identified, of which cases involving individuals under the age of 18 accounted for only 24% (9 out of 38). Details are shown in Table 1.

**Table 1:** Thirty-nine cases of vulvar verruciform xanthoma.

Case	Author	Reported year	Reported nation	Age (years)	Site	Duration	Size (cm)	Morphology	Color	Associated condition	Therapy	Recurrence
# 1	Santa Cruz and Martin (3)	1979	America	12	Vulva	17 years	NA	Multiple verrucous, inverted lesions	NA	NR	NA	No
#2	Santa Cruz and Martin (3)	1979	America	43	Clitoris	NA	1.3	Hyperker, cauliflower-like, verrucous, well-demarcated lesion	Grayish white	Lichen sclerosus	NA	NA
# 3	de Rosa et al. (4)	1989	Italy	65	Vulva	NA	1.5	Plaque	NA	Leiomyomatosis of the uterine corpus	Excisional biopsy	No
# 4	Zamora-Martinez et al. (5)	1990	NA	15	External genitalia	childhood	NA	Soft, exudating	Pink	CHILD	NA	No
# 5	Lonsdale (6)	1992	America	44	Labia majora	1 month	0.3–0.4	Scaly plaque	Pink	NR	Excisions	No
#6	Lu et al. (7)	1994	China	16	Vulva	4 years	About 1.0	Papilliform	NA	NA	NA	NA
# 7	Daimaru et al. (8)	1997	Japan	NA	Vulva	NA	NA	NA	NA	NA	Surgical resection	No
# 8	Kishimoto et al. (9)	1997	Japan	46	Labia majora	Several years	3.5*2.4*2.2	Soft, pedunculated nodule with a new granular lesion	Brown	Fibroepithelial polyp	NA	No
# 9	Hashimoto et al. (10)	1998	Columbia	8	Labia majora	NA	NA	Thick verrucous lesion	Brown	CHILD	NA	NA
# 10	Leong and Meredith (11)	1998	England	84	Left vulva	NA	NA	Verrucous lesion	NA	NR	NA	No
# 11	Reich and Regauer (12)	2004	Austria	38	Labia minora	NA	5.0	Wart-like	Red	NR	NA	Yes
# 12	Reich and Regauer (12)	2004	Austria	30	Labia minora	NA	2.5	Poly	Red	NR	NA	No
#13	Wang et al. (13)	2007	China	47	Vulva	6 months	3*1.5*1	Papule	Pink	No	Surgical resection	NA
#14	Xiang et al. (14)	2007	China	30	Labia majora	1 month	2.0	Cauliflower-like	Grayish yellow	No	Surgical resection	NA
#15	Xiang et al. (14)	2007	China	81	Vulva	1 years	1.0	Verrucous lesion	Grayish yellow	No	Surgical resection	NA
#16	Zhang et al. (15)	2009	China	33	Vulva	32 years	NA	Papules	NA	NA	NA	NA
# 17	Fite et al. (16)	2011	France	51	Labia minora	NA	NA	Verrucous lesion, indurated	Yellow–Orange	Lichen planus	NR	No
# 18	Fite et al. (16)	2011	France	51	Clitoris	NA	0.4	Plaque	Yellow–Orange	Lichen sclerosus	NR	NR
#19	Fite et al. (16)	2011	France	57	Labia minora	NA	2	Multiple indurated plaques	Yellow–Orange	Lichen sclerosus	NR	NR
#20	Fite et al. (16)	2011	France	63	Labia minora	NA	0.5	Indurated plaque	Yellow–Orange	Lichen sclerosus	NR	NR
#21	Fite et al. (16)	2011	France	73	Labia majora	NA	0.4	Leucoplasia	Yellow–Orange	Lichen sclerosus	NR	No
#22	Fite et al. (16)	2011	France	75	Fourchette	NA	1	Indurated plaque	Yellow–Orange	Lichen sclerosus	NR	NR
#23	Fite et al. (16)	2011	France	77	Clitoris	NA	0.2	Indurated plaque	Yellow–Orange	Lichen sclerosus	NR	NR
#24	Fite et al. (16)	2011	France	77	Labia majora	NA	1.5	Indurated plaque	Yellow–Orange	Lichen planus	NR	NR
#25	Fite et al. (16)	2011	France	79	Fourchette	NA	0.3	Indurated plaque	Yellow–Orange	Radiodermatitis	NR	NR
#26	Fite et al. (16)	2011	France	80	Labia majora	NA	0.2	Keratotic papule	Yellow–Orange	Vulvar Paget Disease	NR	NR
#27	Guo and Geng (17)	2012	China	2	Labia majora	1 year	7*5	Multiple neoplasms	Pink	No	Drug treatment (mivamotrexate)	NR
# 28	Frankel et al. (18)	2012	NA	16	Labia majora	9-12 M	1.5	Gran, verrucous	White	NR	NR	No
# 29	Guo et al. (19)	2013	China	1	Labia minora	1 year	7*5	Verrucous plaque	Yellow	NR	NR	NR
# 30	Gantner et al. (20)	2014	Germany	27	Left vulva	Since childhood	NA	Ver hyperker	Red	CHILD	NR	Yes
# 31	Xu et al. (21)	2015	China	5	Vulva	6 years	6*6	Verrucous lesion	Yellow	CHILD	NR	No
# 32	Ijichi et al. (22)	2016	Japan	48	Vulva	2 years	NA	Verrucous surf, well-demarcated	Orange–Red	Severe lymphedema, lymphangioma circumscriptum	NR	No
#33	Cao et al. (23)	2018	China	58	Labia minora	Over 4 years	1.2*0.6	Lichenification	Pink	No	Surgical resection	NR
#34	Feng et al. (24)	2018	China	60	Labia minora	Over 3 years	1.5–2.0	Cauliflower-like	White	No	Surgical resection	No
#35	Zhao et al. (25)	2019	China	22	Vulva	NA	About 1.0	Nodule	Light red	No	Surgical resection	No
#36	Chen et al. (26)	2019	China	20	Labia	6 years	1–3	Puffiness	Yellowish-red	No	Surgical resection	No
#37	Xia and Jiang (27)	2021	China	39	Labia majora	33 years	About 1.0	Verruciform	Pink	No	Surgical resection	No
#38	He et al. (28)	2024	China	12	Left labia majora	over 10 years	6*4	Lobulated plaque	Red	No	Surgical resection	No
# 39	This case	2026	China	12	Perineum	8 years	10*5	Cauliflower-like	Pink	No	Surgical resection	No

## Discussion

VX is a rare benign verrucous and papillary proliferative lesion that occurs on the skin and mucous membranes. The disease was first identified in and named after the oral cavity (29). Its etiology and pathogenesis remain unclear and may be associated with factors such as chronic inflammation, local irritation or recurrent trauma, human papillomavirus (HPV) infection, immune disorders, and genetic factors (1). Clinically, the disease can occur in individuals of all ages, with a male-to-female ratio of approximately 1.2:1. Approximately 80% of cases occur in the oral mucosa, while 20% involve the vulva and skin (1), with the penis, scrotum, anus, and vulva being more commonly affected sites. The lesion is often solitary, while multiple or multi-site lesions are uncommon. Concurrent involvement of the oral cavity, vulva, and skin is rare. However, among the reported cases, the incidence of multiple lesions appears to be increasing (2). The case presented in this study is one of multiple lesions, with the lesions appearing papillary, verrucous, or cauliflower-like, and with colors ranging from flesh-colored or yellowish-brown to light red (30). The disease duration varies.

Due to the low incidence of VX in the female perineal region, particularly its rarity in children, the condition is highly prone to clinical confusion with other conditions such as condyloma acuminatum and vulvar intraepithelial neoplasia (30). Diagnosis based solely on clinical manifestations is challenging, and definitive diagnosis still relies on histopathological examination (31).

Ultrasound examination, as the most commonly used auxiliary diagnostic tool for detecting skin masses (32), offers advantages such as simplicity, non-invasiveness, and affordability. These examinations can reveal the skin and subcutaneous soft tissue layers involved in vulvar VX lesions and assess the internal blood supply, aiding in the differentiation between benign and malignant conditions. Unfortunately, there have been no published reports on VX in the field of ultrasound, nor is there any expert consensus on its ultrasonographic features.

This study attempted to summarize and analyze the ultrasonographic features of this rare case. In combination with previously published research on VX, this study found that the ultrasonographic findings in this patient were highly consistent with histopathological manifestations. Microscopically, a large number of foam cells (12) (macrophages that have phagocytized lipids, also known as xanthoma cells) were observed aggregated in the papillary dermis, accompanied by marked lymphocytic infiltration and a small number of neutrophils. Accordingly, the sonogram presented a hypoechoic lesion. Microscopically, significant necrosis of keratinocytes within the epidermis (33) and the formation of cholesterol crystals were noted, which convincingly explained the multiple punctate hyperechoic foci detected within the lesion on ultrasound. Microscopic examination also demonstrated proliferation, dilation, and congestion of blood vessels in the superficial dermis (34). Two-dimensional ultrasound showed dilated tubular echoes within the solid lesion. CDFI confirmed these tubular structures as vascular, and the lesion demonstrated extremely abundant blood flow signals, flashing in a pattern resembling fireworks. The Alder grade (35) was III, and pulsed wave (PW) Doppler showed a low-velocity, low-resistance flow pattern.

This study represents only a summary and analysis of the ultrasonographic features of a single case. Findings derived from a single case are not reliable, valid, or generalizable. Currently, experience in the ultrasound diagnosis of vulvar VX remains insufficient, and more cases are needed for discovery and synthesis. Additional relevant cases may be collected in the future to further characterize the ultrasonographic features of VX, improve diagnostic accuracy, and provide stronger support for preoperative diagnosis.

Clinically, vulvar VX must be differentiated from other skin conditions such as genital warts, verrucous carcinoma (VC), and xanthomatosis (36). Genital warts (37) are caused by HPV infection, typically occurring on the labia majora and labia minora in females and, rarely, in the oral cavity. They present as pale, light red, or dirty-gray proliferative warts at the infected site, with histopathology revealing characteristic koilocytes. VC is a rare, verrucous, well-differentiated squamous cell carcinoma that most commonly occurs in the external genitalia and oral cavity. It is clinically difficult to distinguish from verruca vulgaris. Pathological examination reveals that cells in VC exhibit atypia, whereas cells in verruca vulgaris show no atypia (38). Xanthomatosis (39) is caused by the deposition of lipids within cells and in the dermis, manifesting as orange–yellow or brown–yellow, smooth-surfaced, tumor-like skin lesions. Its pathological presentation is similar to that of VX, with foam cells visible in both cases. However, in xanthomatosis, foam cells can appear in the dermis, subcutaneous tissue, and tendons, whereas in VX, foam cells are confined to the papillary dermis.

As one of the common auxiliary diagnostic tools for skin masses (40), ultrasound examination enables risk stratification for tumor invasiveness through a comprehensive analysis of growth patterns and the layers involved, thereby assisting in distinguishing lesions with similar appearances (41). Certain malignant skin tumors exhibit characteristic ultrasonographic features. For example, basal cell carcinoma may demonstrate scattered or clustered punctate hyperechoic foci (42, 43). Additional malignant signs detectable on ultrasound include focal disruption of epidermal continuity, irregular depression of the lesion’s anterior margin, and indistinct borders between the lesion base and adjacent tissues (indicating invasion of deeper structures) (44). Increased intralesional blood flow, elevated flow velocity, and multiple vascular pedicles at the lesion base may be suggestive of malignancy (45). Moreover, preoperative ultrasound examination can further evaluate regional lymph nodes and other organs (46) to detect possible metastases, thereby aiding in the differentiation between benign and malignant skin masses.

Currently, there is no standardized treatment protocol for this disease, and management is primarily symptomatic. Complete surgical excision remains the main treatment modality for vulvar VX (25), with a favorable prognosis. However, cases involving extensive lesions remain challenging to manage and are associated with significant psychological distress for patients. Preoperative ultrasound examination allows for the assessment of the extent of lesion involvement, precisely guiding clinicians in planning surgical excision margins and avoiding extensive excisions. Recurrent cases have been reported in the literature (12), and accurate delineation of the lesion extent using ultrasound may help reduce the recurrence rate to some degree. Furthermore, ultrasound can serve as an important follow-up tool for evaluating potential recurrence in patients.

In conclusion, the possibility of vulvar VX should be considered when verrucous lesions appear on or around the vulva. A definitive diagnosis of vulvar VX must be confirmed through histopathological examination, and ultrasound can serve as an important auxiliary tool for differential diagnosis. The potential ultrasonographic features of vulvar VX include a hypoechoic dermal mass with multiple punctate calcifications and extremely abundant internal blood flow. Complete surgical excision remains the primary treatment modality for vulvar VX, and ultrasound examination plays a significant role in preoperative guidance for surgical planning and in postoperative follow-up.

## Data Availability

The original contributions presented in the study are included in the article/Supplementary material; further inquiries can be directed to the corresponding authors.
