# Supplementary material for: Giant vulvar verruciform xanthoma in a child: a rare case report and literature review
Source: Front Med (Lausanne). 2026 Apr 22;13:1774843. doi: 10.3389/fmed.2026.1774843 (PMC13148186; doi:10.3389/fmed.2026.1774843)
Supplement: Supplementary file 1 [file Table_1.docx]

| Case | Author | Reported Year | Reported Nation | Age  (years) | Site | Duration | Size  (cm) | Morphology | Color | Associated condition | Therapy | Recurrence |
| --- | --- | --- | --- | --- | --- | --- | --- | --- | --- | --- | --- | --- |
| # 1 | Santa Cruz DJ (3) | 1979 | America | 12 | Vulva | 17 years | NA | Multiple verrucous, inverted lesion | NA | NR | NA | No |
| #2 | Santa Cruz DJ (3) | 1979 | America | 43 | Clitoris | NA | 1.3 | Hyperker, cauliflower-likel, verrucous, well-demarcated lesion | Grayish white | Lichen sclerosus | NA | NA |
| # 3 | de Rosa G (4) | 1989 | Italy | 65 | Vulva | NA | 1.5 | Plaque | NA | leiomyomatosis of uterine corpus | Excisional biopsy | No |
| # 4 | Zamora-Martinez E (5) | 1990 | NA | 15 | External genitalia | childhood | NA | Soft, exudating | Pink | CHILD | NA | No |
| # 5 | Lonsdale RN (6) | 1992 | America | 44 | Labia majora | 1 month | 0.3-0.4 | Scaly plaque | Pink | NR | Excisions | No |
| #6 | Lu XM (7) | 1994 | China | 16 | Vulva | 4 years | About 1.0 | Papilliform | NA | NA | NA | NA |
| # 7 | Daimaru Y (8) | 1997 | Japan | NA | Vulva | NA | NA | NA | NA | NA | Surgical resection | No |
| # 8 | Kishimoto S (9) | 1997 | Japan | 46 | Labia majora | Several years | 3.5*2.4*2.2 | Soft, pedunculated nodule with new granular lesion | Brown | fibroepithelial polyp | NA | No |
| # 9 | Hashimoto K (10) | 1998 | Columbia | 8 | Labia majora | NA | NA | Thick verrucous lesion | Brown | CHILD | NA | NA |
| # 10 | Leong FJ (11) | 1998 | England | 84 | Left vulva | NA | NA | Verrucous lesion | NA | NR | NA | No |
| # 11 | Reich O (12) | 2004 | Austria | 38 | Labia minora | NA | 5.0 | Wart-like | Red | NR | NA | Yes |
| # 12 | Reich O (12) | 2004 | Austria | 30 | Labia minora | NA | 2.5 | Poly | Red | NR | NA | No |
| #13 | Wang S (13) | 2007 | China | 47 | Vulva | 6 months | 3*1.5*1 | Papule | Pink | No | Surgical resection | NA |
| #14 | Xiang JJ (14) | 2007 | China | 30 | Labia majora | 1 month | 2.0 | Cauliflower-like | Grayish yellow | No | Surgical resection | NA |
| #15 | Xiang JJ (14) | 2007 | China | 81 | Vulva | 1 years | 1.0 | Verrucous lesion | Grayish yellow | No | Surgical resection | NA |
| #16 | Zhang J (15) | 2009 | China | 33 | Vulva | 32 years | NA | Papules | NA | NA | NA | NA |
| # 17 | Fite C (16) | 2011 | France | 51 | Labia minora | NA | NA | Verrucous lesion, indurated | Yellow-Orange | lichen planus | NR | No |
| # 18 | Fite C (16) | 2011 | France | 51 | Clitoris | NA | 0.4 | Ind plaque | Yellow-Orange | lichen sclerosus | NR | NR |
| #19 | Fite C (16) | 2011 | France | 57 | Labia minora | NA | 2 | Multiple Indurated plaques | Yellow-Orange | lichen sclerosus | NR | NR |
| #20 | Fite C (16) | 2011 | France | 63 | Labia minora | NA | 0.5 | Indurated plaque | Yellow-Orange | lichen sclerosus | NR | NR |
| #21 | Fite C (16) | 2011 | France | 73 | Labia majora | NA | 0.4 | Leucoplasia | Yellow-Orange | lichen sclerosus | NR | No |
| #22 | Fite C (16) | 2011 | France | 75 | Fourchette | NA | 1 | Indurated plaque | Yellow-Orange | lichen sclerosus | NR | NR |
| #23 | Fite C (16) | 2011 | France | 77 | Clitoris | NA | 0.2 | Indurated plaque | Yellow-Orange | lichen sclerosus | NR | NR |
| #24 | Fite C (16) | 2011 | France | 77 | Labia majora | NA | 1.5 | Indurated plaque | Yellow-Orange | lichen planus | NR | NR |
| #25 | Fite C (16) | 2011 | France | 79 | Fourchette | NA | 0.3 | Indurated plaque | Yellow-Orange | radiodermatitis | NR | NR |
| #26 | Fite C (16) | 2011 | France | 80 | Labia majora | NA | 0.2 | Keratotic papule | Yellow-Orange | Vulvar Paget Disease | NR | NR |
| #27 | Guo YY (17) | 2012 | China | 2 | Labia majora | 1 year | 7*5 | Multiple neoplasms | Pink | No | Drug treatment (mivamotrexate) | NR |
| # 28 | Frankel MA (18) | 2012 | NA | 16 | Labia majora | 9-12M | 1.5 | Gran, verrucous | White | NR | NR | No |
| # 29 | Guo Y (19) | 2013 | China | 1 | Labia minora | 1 year | 7*5 | Verrucous plaque | Yellow | NR | NR | NR |
| # 30 | Gantner S (20) | 2014 | Germany | 27 | Left vulva | Since childhood | NA | Ver hyperker | Red | CHILD | NR | Yes |
| # 31 | Xu XL (21) | 2015 | China | 5 | Vulva | 6 years | 6*6 | Verrucous lesion | Yellow | CHILD | NR | No |
| # 32 | Ijichi A (22) | 2016 | Japan | 48 | Vulva | 2 years | NA | Verrucous surf, well-demarcated | Orange-Red | Severe lymphedema, lymphangioma circumscriptum | NR | No |
| #33 | Cao YJ (23) | 2018 | China | 58 | Labia minora | Over 4 years | 1.2*0.6 | Lichenification | Pink | No | Surgical resection | NR |
| #34 | Feng L (24) | 2018 | China | 60 | Labia minora | Over 3 years | 1.5-2.0 | Cauliflower-like | White | No | Surgical resection | No |
| #35 | Zhao SL (25) | 2019 | China | 22 | Vulva | NA | About 1.0 | Nodule | Light red | No | Surgical resection | No |
| #36 | Chen YY (26) | 2019 | China | 20 | Labia | 6 years | 1-3 | Puffiness | Yellowish-red | No | Surgical resection | No |
| #37 | Xia JL (27) | 2021 | China | 39 | Labia majora | 33 years | About 1.0 | Verruciform | Pink | No | Surgical resection | No |
| #38 | Meihua He (28) | 2024 | China | 12 | Left labia majora | over 10 years | 6*4 | Lobulated plaque | Red | No | Surgical resection | No |
| # 39 | This case | 2026 | China | 12 | Perineum | 8 years | 10*5 | Cauliflower-like | Pink | No | Surgical resection | No |
